# Lifestyle Disease Surveillance Using Population Search Behavior: Feasibility Study

**DOI:** 10.2196/13347

**Published:** 2020-01-27

**Authors:** Shahan Ali Memon, Saquib Razak, Ingmar Weber

**Affiliations:** 1 Language Technologies Institute School of Computer Science Carnegie Mellon University Pittsburgh, PA United States; 2 Carnegie Mellon University Doha Qatar; 3 Social Computing Department Qatar Computing Research Institute Hamad Bin Khalifa University Doha Qatar

**Keywords:** noncommunicable diseases, lifestyle disease surveillance, infodemiology, infoveillance, Google Trends, Web search, nowcasting, public health, digital epidemiology

## Abstract

**Background:**

As the process of producing official health statistics for lifestyle diseases is slow, researchers have explored using Web search data as a proxy for lifestyle disease surveillance. Existing studies, however, are prone to at least one of the following issues: ad-hoc keyword selection, overfitting, insufficient predictive evaluation, lack of generalization, and failure to compare against trivial baselines.

**Objective:**

The aims of this study were to (1) employ a corrective approach improving previous methods; (2) study the key limitations in using Google Trends for lifestyle disease surveillance; and (3) test the generalizability of our methodology to other countries beyond the United States.

**Methods:**

For each of the target variables (diabetes, obesity, and exercise), prevalence rates were collected. After a rigorous keyword selection process, data from Google Trends were collected. These data were denormalized to form spatio-temporal indices. L1-regularized regression models were trained to predict prevalence rates from denormalized Google Trends indices. Models were tested on a held-out set and compared against baselines from the literature as well as a trivial last year equals this year baseline. A similar analysis was done using a multivariate spatio-temporal model where the previous year’s prevalence was included as a covariate. This model was modified to create a time-lagged regression analysis framework. Finally, a hierarchical time-lagged multivariate spatio-temporal model was created to account for subnational trends in the data. The model trained on US data was, then, applied in a transfer learning framework to Canada.

**Results:**

In the US context, our proposed models beat the performances of the prior work, as well as the trivial baselines. In terms of the mean absolute error (MAE), the best of our proposed models yields 24% improvement (0.72-0.55; *P*<.001) for diabetes; 18% improvement (1.20-0.99; *P*=.001) for obesity, and 34% improvement (2.89-1.95; *P*<.001) for exercise. Our proposed across-country transfer learning framework also shows promising results with an average Spearman and Pearson correlation of 0.70 for diabetes and 0.90 and 0.91 for obesity, respectively.

**Conclusions:**

Although our proposed models beat the baselines, we find the modeling of lifestyle diseases to be a challenging problem, one that requires an abundance of data as well as creative modeling strategies. In doing so, this study shows a low-to-moderate validity of Google Trends in the context of lifestyle disease surveillance, even when applying novel corrective approaches, including a proposed denormalization scheme. We envision qualitative analyses to be a more practical use of Google Trends in the context of lifestyle disease surveillance. For the quantitative analyses, the highest utility of using Google Trends is in the context of transfer learning where low-resource countries could benefit from high-resource countries by using proxy models.

## Introduction

### Background and Prior Work

Public health surveillance is the systematic collection, analysis, and interpretation of health-related data to be used by those responsible for preventing and controlling disease and injury [1]. One of the most common examples of public health surveillance involves what is known as *disease surveillance*. Disease surveillance is traditionally accomplished through a system of manual surveys, or mandatory reporting by the doctors to the government. However, such a system is costly, prone to missing new and rare events, and has a high time lag. Hence, in the past decade, there has been an increase in the use of Web-based data for disease surveillance with the goal of supplementing, not replacing, traditional methods.

One of the first applications of Web-based disease surveillance was the tracking of influenza using Web-based search behavior. A seminal study on this was carried out by Ginsberg et al [2] in the creation of Google Flu Trends (GFT). The purpose of GFT was to monitor health-seeking behavior by analyzing Google search queries to track influenza-like illness (ILI) in a population. Although, the system shut down in 2015 for overestimating the influenza epidemics [3], it started a whole line of research using Google Trends to nowcast anything from US presidential elections [4], to fertility rates [5], stock prices [6], box office sales [7], and other economic indicators [8]. But more than anything, it set a precedent for using Web search data for nowcasting and monitoring diseases [9].

Most of the work on disease surveillance focuses on *fast-moving* infectious or communicable diseases where the goal is to predict the disease outbreak as early as possible [10]. In this domain, previous work includes using data for predicting influenza in South China [11], using Google Trends to determine relationship between sexually transmitted infection (STI)–related search engine trends and STI rates [12], and the use of other search engines such as Yahoo for surveillance of influenza in the United States [13].

However, noncommunicable diseases (NCDs) or lifestyle diseases, such as obesity, smoking, diabetes, depression, or lack of physical activity, account for a far larger share of both the US and global health care system’s cost. According to the World Health Organization, lifestyle diseases are responsible for around 70% of all deaths globally every year [14]. In the United States, the economic burden of obesity-related diseases alone is around US $190 billion [15]. This makes tracking of these diseases important for timely allocation of resources and implementation of interventions such as taxation, policy changes, or public health campaigns. However, the current traditional methods of surveillance used in the United States are costly, labor-intensive, and time-consuming. In the international setting, most countries do not even have such surveillance systems, and lack data and statistics about population behavior and disease risk factors. This has triggered researchers to use data on the Web, and specifically Web search activity as a proxy to predict the prevalence of NCDs.

Google Trends is one of the most popular tools for analyzing Web search activity. Research in the domain of using Google Trends covers anything from the prediction of suicide risks, depression, shared migraine experiences, and stress in a population [16-21] to monitoring of nonsuicidal self-injury rates [22], correlational studies between Google Trends and actual suicide risk and rates [23-27], influence of seasons on the incidences of depression [28], study of psychological and social factors affecting internet searches related to suicide [29], seasonality in seeking of mental health information [30], low validity of Google Trends in forecasting suicidal risk [31], the infoveillance of cancer incidence rates [32,33], mortality rates [34], obesity [35], diabetes [36], dental caries [37-39], the behavioral forecasting and awareness of alcohol consumption rates, and drugs [17], seasonal variation in ophthalmology-related diseases [40], geographical variance in stroke prevalence [41], and a multitude of other relevant literature, a survey of which can be found in [9].

### Existing Challenges in Modeling Noncommunicable Diseases With Google Trends Data

As illustrated above, the literature on the infodemiology and infoveillance of NCDs is extensive and diverse. However, almost all of it suffers from a fundamental problem when trying to build models—the scarcity of data. This is primarily a consequence of the *slow-moving* nature of most of the NCDs, as well as the lack of resources to conduct finer temporal surveillance. As a result, the surveillance data for lifestyle diseases are typically available on an annual basis as opposed to weekly basis for ILI. It is important to note that even if the finer temporal surveillance was possible, owing to the *slow-moving* nature of the NCDs, there would not be discernible changes in the data points to conduct any useful analysis as, for example, obesity rates are unlikely to change week over week. Notwithstanding the data scarcity issues in the temporal surveillance, most of the background literature still falls back on using time-series data to do some version of correlational studies. These analyses span across countries, states, cities, or metro areas. However, because Google Trends data are only available starting 2004, *most* of the temporal correlational studies for NCDs will have only as many data points as years passed since 2004: a maximum of 15 data points for a given location. This results in nonrobust time-series-based approaches as they are subject to overfitting. Another major limitation of these studies is the ad-hoc keyword selection. More concretely, most of the aforementioned literature resorts to using hand-picked keywords as features for the target variable at hand. This introduces an a priori bias and subjectivity in the predictive system, as well as the evaluation.

To deal with the first problem on the limitations of the temporal data, a few studies have tried to use state-level data to fit US national-level trends. In this regard, a relevant research was carried out by Sarigul and Rui [35] on nowcasting obesity. They used manually selected terms and their correlations to predict regional obesity prevalence by modeling regional as well as temporal variations in the Google Trends data. However, their methods use ad-hoc keyword selection and tend to use a fitting approach without predictive out-of-sample evaluations.

Another relevant study has used spatial data to predict temporal trends. This research was carried out by Nguyen et al [42] and uses linear regression model along with Lasso regularization, modeling regional variation for different NCDs to predict prevalence by state for a particular target year. One novel contribution of their study is the semiautomated keyword selection process using semantically related terms as keywords. Unlike other studies, they perform an out-of-sample evaluation. However, one of their key shortcomings is the lack of an appropriate denormalization model as explained as follows: Although they train a model across space, they, then, apply it across time, without accounting for the fact that, within a given year, the spatial data are independently normalized by Google. Hence, although the individual spatial trends might be appropriate to track with these data, the national-level model would miss out on national trends in time. As an example, if the national search intensity for terms predictive of NCDs was to double from 2014 to 2015 with the relative spatial distribution remaining constant, then spatial data alone would not pick up such a temporal trend. In fact, it would treat 2014 exactly as 2015. To account for such national temporal variations, Phillips et al’s study on the relationship between state-level search behavior and the cancer incidence in the United States [32] uses time in years as a continuous covariate to control for temporal trends. However, due to the fundamental intricacies of the way Google normalizes its data, discussed in the next section, such an approach is not likely to generalize.

A very small subset of the background literature [30,31,37-40,43] explicitly tests the application of their models to other countries or geographic regions. In essence, most of the previously proposed surveillance models or techniques remain inconclusive in their generalizability to other spatial reference frames. To correct for this, we explore the utility of our approach in the international setting by using a transfer learning framework.

Finally, *all* of the literature on the surveillance of NCDs using Google Trends leaves out obvious yet important evaluation criteria: a comparison of their results to the trivial *last year equals this year* baseline. This is going to be one of the key themes of this paper while we evaluate the validity of Google Trends to predict the prevalence of NCDs.

A brief summary of the comparison of previous literature on the lifestyle disease surveillance using Google Trends in relation to our contribution across several metrics is presented in Table 1. The first column represents the literature surveyed; the second column represents if the literature used any sort of automation in terms of keyword selection; the third column surveys the type of data used in the study; the fourth column represents the inclusion of data denormalization if applicable; the fifth column surveys the type of evaluation used; the sixth column shows if the study compared its evaluation with a trivial baseline; the seventh column describes the geographical setting the study was based on; and the eighth column describes if any secondary evaluation of the proposed methodology is shown for a different geographical setting. In terms of the cell values, *x* represents missing; *N/A* represents not applicable; and *✓* represents available.

**Table 1:** A survey and comparison of previous literature across different metrics.

Studies	Bootstrapping keyword selection	GT data type	Data denormalization	Predictive evaluation	Comparison to trivial baseline	Geographical setting	Generalizability to other geographical setting
Leffler et al [40]	x^a^	Temporal	N/A^b^	In-sample	N/A	United States, the United Kingdom, Canada, and Australia	✓^c^
Yang et al [28]	x	Temporal	N/A	In-sample	N/A	Worldwide	x
McCarthy [18]	x	Temporal	N/A	In-sample	x	United States	x
Hagihara et al [24]	x	Temporal	N/A	In-sample	N/A	Japan	x
Sueki [25]	x	Temporal	N/A	In-sample	N/A	Japan	x
Walcott et al [41]	x	State-level	N/A	In-sample	N/A	United States	x
Yang et al [23]	x	Temporal	N/A	In-sample	N/A	Taipei City, Taiwan	x
Ayers [30]	x	Temporal	N/A	In-sample	N/A	United States, and Australia	✓
Bragazzi [22]	x	Temporal	N/A	In-sample	N/A	Italy	x
Braun and Harréus [44]	x	Temporal	N/A	In-sample	N/A	Germany	x
Breyer and Eisenberg [36]	x	Temporal	N/A	In-sample	N/A	United States	x
Gunn III and Lester [21]	x	State-level	N/A	In-sample	x	United States	x
Ingram Plante [43]	x	Temporal	N/A	In-sample	N/A	United States, Australia, Germany, the United Kingdom, and Canada	✓
Willard and Nguyen [45]	x	State-level	N/A	In-sample	N/A	United States	x
Bragazzi [46]	x	Temporal	N/A	In-sample	x	Italy	x
Brigo et al [47]	x	Temporal	N/A	In-sample	N/A	Worldwide	x
Bruckner et al [26]	x	Temporal	N/A	In-sample	N/A	England and Wales	x
Sarigul et al [35]	x	State-level	x	In-sample	x	United States	x
Song et al [29]	x	Temporal	N/A	In-sample	N/A	Korea	x
Nguyen et al [42]	✓	State-level	x	Out-of-sample	x	United States	x
Wang et al [48]	✓	Temporal	N/A	In-sample	x	Taiwan	x
Ma-Kellams et al [19]	x	State-level	N/A	In-sample	x	United States	x
Parker et al [17]	x	State-level	x	Out-of-sample	x	United States	x
Burns et al [16]	x	Temporal	N/A	In-sample	N/A	United States	x
Cervellin et al [49]	x	Temporal	N/A	In-sample	x	Italy	x
Hassid et al [50]	x	Temporal	N/A	In-sample	N/A	United States	x
Lotto et al [38]	✓	Temporal	x	In-sample	N/A	United States, United Kingdom, Australia, and Brazil	✓
Ojala et al [5]	✓	State-level, temporal	N/A	Out-of-sample	x	United States	x
Ricketts and Silva [34]	x	Temporal	N/A	In-sample	x	United States	x
Tran et al [31]	✓	Temporal	N/A	In-sample	N/A	United States, Germany, Austria, and Switzerland	✓
Wehner et al [33]	x	Temporal	N/A	In-sample	x	United States	x
Aguirre et al [37]	✓	Temporal	x	In-sample	N/A	United States, United Kingdom, Germany, Brazil, France, India, Italy, Japan	✓
Arendt [27]	x	Temporal	N/A	In-sample	x	Worldwide	x
Chandler [20]	x	State-level	x	In-sample	x	United States	x
Coogan et al [51]	x	Temporal	N/A	In-sample	x	Australia	x
Phillips et al [32]	x	State-level	x	In-sample	x	United States	x
Cruvinel et al [39]	✓	Temporal	x	In-sample	N/A	10 South American Countries	✓
*This study* ^d^	✓	*State-level, temporal*	✓	*Out-of-sample*	✓	*United States*	✓

^a^x: missing.

^b^Not applicable.

^c^✓: available.

^d^The values in italics signify how our study compares to those from the past literature across different metrics.

In summary, we identified the following key issues in the aforementioned background literature:

1. Ad-hoc keyword selection.
2. Overfitted temporal analysis.
3. Spatial analysis without appropriate denormalization.
4. Insufficient predictive evaluation.
5. Lack of evidence for generalization to other countries.
6. Failure to compare results to trivial baselines.

### Study Objectives

The insufficient evaluation metrics, and methodological errors in the background review, set out a motivation to validate the use of Google Trends for nowcasting NCDs. Hence, in this study, we had 3 key objectives:

1. To use a corrective approach to first rectify the methodological shortcomings of the previous literature.
2. To study the limitations and promises of Google Trends in the context of its accuracy and robustness to predict national lifestyle disease trends.
3. To experimentally test the generalizability of this approach to other similar countries.

## Methods

### Study Design

The methods we used for our study differ from previous work, as explained above, in (1) how we select search terms for Google Trends to limit cherry picking, (2) how we denormalize Google Trends data to overcome certain limitations, (3) the focus on an out-of-sample evaluation rather than in-sample model fit, (4) the inclusion of a trivial baseline for comparison, and (5) a transfer learning setup to evaluate cross-country generalizability.

### Terminology

To facilitate understanding of the description of our methodology, we have defined a set of key terms that we used frequently in this paper. These terms and their definitions are as follows:

#### Offline Target Variable

This refers to the variable of interest that we are monitoring. In our case, we monitored diabetes, obesity, and exercise.

#### Offline Data

For any offline target variable (such as diabetes), offline data are the actual regional or national prevalence of the condition. Although *offline target variable* refers to the name of the variable we are monitoring—say, *Diabetes*—*offline data* refers to actual numerical values pertaining to that variable—say, US state-level diabetes prevalence rates in 2014.

#### Spatial Data

For the purpose of this paper, spatial data refer to Google Trends’ Web search intensity for a given year and a particular keyword normalized across different US states.

#### Temporal Data

Temporal data refer to Google Trends’ US Web search intensity for a particular keyword normalized across different years.

### Offline Data Collection

For each of the target variables, we collected the offline data across 15 years from 2004 to 2018 each year separately. This includes data for the 50 states (including Washington, DC and excluding Hawaii as offline data for Hawaii were unavailable for the year of 2004). For prevalence rates, we used the Center for Disease Control and Prevention’s Behavioral Risk Factor Surveillance System (BRFSS) [52].

### Keyword Selection

In this phase, we used 3 tools for the keyword selection: (1) Google Correlate, (2) related search queries, and (3) Semantic Link, a Web-based service to find related terms. A subset of the resulting keywords from each of the 3 sources is presented in Table 2.

We bootstrapped the keyword selection process as follows, starting from a set of seed terms.

**Table 2:** The subset of unpruned keywords for different target variables.

Target variable	Google Correlate	Semantic Link	Related Queries
Diabetes	when i get up	insulin	diabetes symptoms
	sell avon	polyphagia	signs of diabetes
	medicine for dogs	ketoacidosis	prediabetes
	very weak	cholesterol	icd 10
	sugar level	hypertension	icd 10 type 2 diabetes
Obesity	catherines.com	abdominal	food delivery near me
	dresses plus size	anorexia	lose fat
	sims 3 games	BMI	myfitnesspal
	lose 100 pounds	appetite	indeed.com
	dresses plus	ADHD	pizza delivery
Exercise	transportation options	exercises	my fitness pal
	best bike	aerobic	workout
	bike laws	jogging	iPod
	bike repair	gyms	quinoa gluten free
	bike frame size	muscles	how to exercise

#### Seed Terms

For each target variable, we used 1 or 2 seed terms. For diabetes, we chose *diabetes* and *diabetic*, for obesity, we chose *obesity* and *obese*, and for exercise, we chose *exercise*. These seed terms were, then, used to generate other cooccurring terms in English Wikipedia using Semantic Link [53] as mentioned in the study by Nguyen et al [42]. Other methods to enhance related terms, such as those described in the study by Lampos et al [54] on using word embeddings could also be used.

#### Google Correlate

Google Correlate is a tool that takes either a temporal or a spatial series as input and returns a ranked list of Web search queries that are correlated across time or space [55]. For our purposes, we used the offline data for the year 2015 to determine top 30 to 40 keywords that strongly correlate across all the US states including Washington, D.C. We, then, pruned many of those keywords as has been described later.

#### Related Search Queries

We used a combination of the keywords selected in the aforementioned methods in Google Trends to output-related search queries.

#### Pruning

One of the most common methods to avoid overfitting is by using dimensionality reduction or by using regularized models. However, these methods do not guard against keywords with spurious correlations. For example, the query *sims 3 games* in Table 2 is one of the top 10 spatially correlated keywords for obesity. A less obvious example is the search term, *catherines.com*. Catherines is a store selling plus-size clothing and so search volume for *catherines.com* has an arguable causal connection to obesity. However, its search volume is also tied to its market share, which can change over time. As such, it might be a robust feature for a spatial-only model, but we decided to remove such *branded* search terms for temporal analysis. Finally, apart from removing nonsensical and branded search terms, we also removed any keywords with low search volume. All these selections are made in an effort to reduce overfitting and increase model robustness across time. A list of all the postpruning keywords used for this study for each of the target variables can be found in Multimedia Appendix 1.

### Google Trends Data Collection

We used Google Trends to collect 2 kinds of data: spatial and temporal. For spatial data, we used each keyword-year combination as a query to Google Trends to get across-state data, that is, 50 data points for each keyword and each of the 15 years, 2004-2018. For temporal data collection, we collected data across the 15 years at the US national level rather than at the state level. This was done to reduce the required data collection effort by a factor of 50. Note that the state-level temporal trends are implicitly collected already as we have (1) state-level relative volumes within a given year, as well as (2) US national-level temporal trends across the years. We have explained how we combine these 2 types of data points to create a spatio-temporal model in following sections.

As Google Trends does not provide an official application programming interface, other than their *export as .csv* option, we made use of Python’s *pytrends* [56] package, which can be used to retrieve data from Google Trends. Regardless of which method is used to obtain the data, one caveat is that Google Trends’ data are not stable and that repeatedly asking for the same data can return different results. Concretely, since Google search volume index is calculated by a sampling method, the results even for historic data can fluctuate day to day [31,57]. To limit such fluctuations and instability in the search data, similar to [31], we sample and average each data point 10 times across time with a gap of a day between each sample for the United States, and 3 times across each sample for Canada.

Another important detail is that Google Trends results for an individual term such as *diabetes* include search phrases such as *diabetes insulin* or *insulin diabetes* [58]. This can create collinearity for the results for different terms, which has to be taken into account when and if doing a post hoc feature analysis.

### Google Trends Data Normalization

For both privacy and business reasons, Google Trends does not show absolute search volumes but only *normalized* search intensity, and it is important to understand the process of this normalization [59]. One of our contributions is a data calibration mechanism, which is based on a proper understanding of the underlying normalization procedure.

Concretely, search intensity is different from absolute search volume in that it measures the *relative* interest in a search term, that is, the fraction of all searches in the reference temporal or spatial unit. One desired consequence of this is that as Google’s user base grows over time, the search intensity does not trivially increase, making it potentially comparable across time.

Another point to understand is that there is a certain interplay between the search terms. For example, if the search volume for the keyword *justin bieber* was to go up 10-fold, with everything else remaining constant, then the *relative* search intensity for other terms would still drop (slightly), even though their *absolute* search volume remains unchanged.

The search intensity is normalized across time or across space depending on the mode of data collection. For example, when collecting temporal data across several years for the query *tomacco*, Google returns data such that the period with the highest relative search intensity corresponds to an arbitrary reference value of 100. All other temporal units are normalized with respect to this absolute maximum of 100, meaning that a value of 30 means that in a corresponding time unit, the relative fraction of searches matching the criteria was only 30% of what it is during the peak. Similarly, when getting search data across spatial units such as US states, the state with the highest search intensity is assigned a value of 100, and all other spatial units are normalized relative to the search intensity of that peak location.

In our setting, we combined data across both space and time. The primary reason behind doing that is to increase the number of training instances in an effort to compensate for the slow-moving nature of the NCDs. This, in turn, helps us to learn a national generalizable model. Combining spatial and temporal data requires undoing Google’s normalization for the following reason: within each year, data are normalized independently across space. Thus, the value of 100 in year 2014 for the state of California cannot be compared with the value of 100 in the year 2015 for the state of Texas. From 2014 to 2015, the overall search volume may have gone up or down, and spatial data alone do not reveal such information. By appropriately combining the spatial data with temporal trends, we are able to effectively undo Google’s normalization to correctly juxtapose the data for 2014 next to the data for 2015 such that a relative increase in numbers actually corresponds to a relative increase in search intensity.

**Figure 1:**
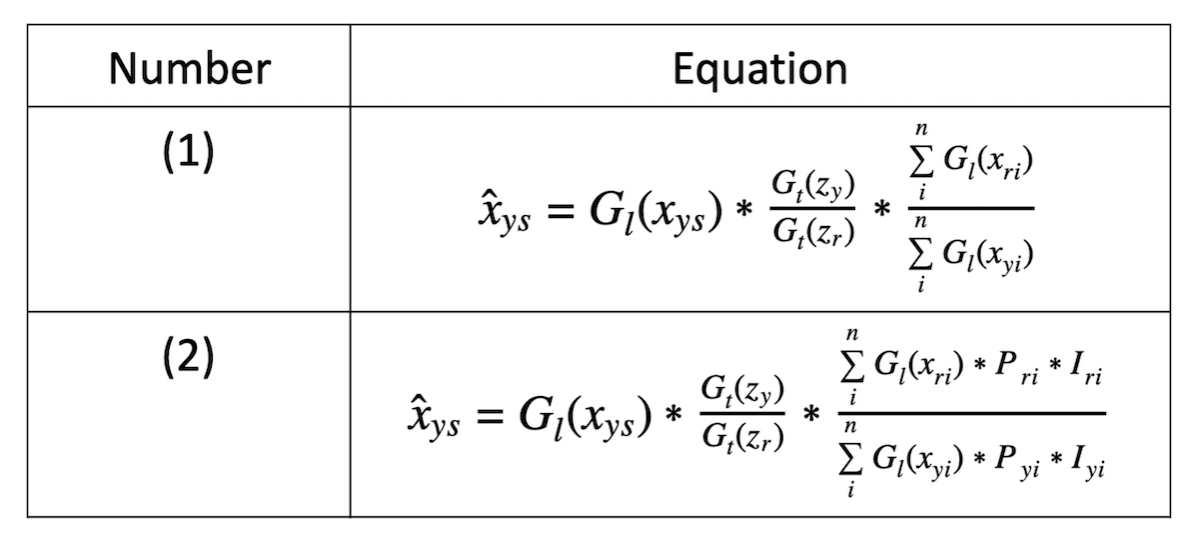
The equations for the proposed denormalization framework.

To reconstruct the state-level contribution to the national value, we need to take into account the actual absolute search volume from each of the states. As those data are not available to us, we approximated that using the following 2 steps:

(1) To undo the effect of spatial and temporal normalizations, we chose 2004 as a reference year *r* to rescale and denormalize year-state values as in equation (1) in Figure 1.

Here 
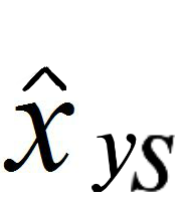
 is the denormalized scalar value for year *y* and state *s*. Furthermore, *G* signifies that the data were collected via Google Trends, *G_l_* represents that data are spatial (where *l* stands for location), that is, normalized across the US states by Google, represents the year, *s* represents the state, and *G_l_ (x_ys_)* represents a single state-level data point for the year *y* obtained from Google Trends. *G_t_* represents that data are temporal (where *t* stands for time), *G_t_ (z_y_)* represents the value of the corresponding keyword at the national level in year *y* across time, that is, normalized across the different years, *G_t_ (z_r_)* represents the across-time value of the corresponding keyword in the reference year *r*, where for our purposes, *r=2004. ∑^n^_i_ G_l_ (x_ri_)* represents the sum of the regional distribution of the corresponding keyword in the reference year 2004 where *n* is the number of states. *∑^n^_i_ G_l_ (x_yi_)* represents the sum of the regional distribution of the corresponding keyword in the year y.

(2) Another important insight to realize is that different regions in the United States contribute differently to the US national trends because of differences in absolute search volume. Regions with large populations, and large numbers of Google search users, such as California or New York, will have more of an impact on the national trend than regions with small populations. However, the relative search intensities normalize for different numbers of issued Google searches. Hence, to debias our data on the population level, after following step 1, we adjusted each value by a product of the population in each state multiplied by the internet penetration to get an approximate number of Google search users in each state. We collected the internet penetration rates from the BRFSS site [52]. Note that we do not need to have an absolute number of Google users. For our method to work correctly, all that matters is that we have a relative multiple of that (unknown) number.

As both state-level populations and internet penetration can change over time, ideally, we would want to use different correction factors for each year. However, as we observed that both population sizes and internet penetration rates increased fairly uniformly across all states, the correction factors for different years were correlated at the level of *approximately .99*. For this reason, we chose to apply only a single, static, set of state-level correction factors from the year 2015.

After following the denormalization procedure, we were left with a matrix where each year-state value can be compared with each other year-state value in a meaningful manner. This allowed us to, effectively, multiply our training data across different years and different states.

With steps 1 and 2, our final formula was as shown in equation (2) in Figure 1, where all the terms are same as the equation (1). *P_ri_* represents the population size for the reference year *r* and state *i*. *P_yi_* represents the population size for the year *y* and state *i*. *I_ri_* represents the internet penetration for the reference year *r* and state *i*. *I_yi_* represents the internet penetration for the year *y* and state *i*

Note that the final output *x̂_ys_* is a scalar, indexed by both year *y* and US state *s*.

For an example-based explanation of the denormalization process, see Multimedia Appendix 2 [60].

### Regression Modeling

Even after our approach for obtaining our year-state data matrix, we did not have sufficient data to fit complex prediction models such as deep neural networks [61]. Hence, we fit (regularized) linear regression models to predict slow-moving trends such as diabetes, obesity, and exercise rates for the 50 states in the United States. We used Python’s scikit [62] library to fit our linear regression models. Concretely, given *y* as the ground truth and 
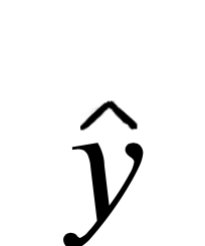
 as the prediction*,* we fit a model of the form:


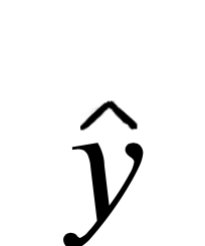
^=wx+b (1


by minimizing the loss *L*


L=(
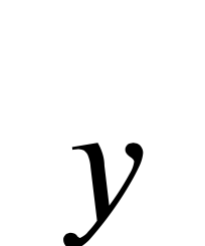
−
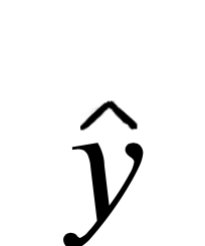
)^2^+λ
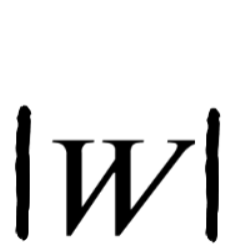
 (2)


where *x* represents the feature set, *w* represents the unknown parameters, and *b* represents the bias.

To avoid overfitting, and to yield a simpler and interpretable model, we used Lasso [63] as the shrinkage, which combines ordinary least-square regression with an L-1 regularization. We also experimented with Ridge regression (without consulting the testing set) [64] but the cross-validated performance on the training set was similar, and Lasso regression gave a sparser model. Lasso requires a regularization parameter *λ* to govern the trade-off between more complex models with better performance on the test data (=small *λ*) and simpler models with potentially better performance on unseen data (=larger *λ*).

In this paper, the optimal *λ* was determined by using k-fold cross validation, optimizing for the negative mean squared error. Feature values were standardized to have zero mean and unit variance. For our purposes, we used *k=12* corresponding to the number of years in the training set (2005-2016).

### Training, Validation, and Testing Phase

For the training phase, we used data from 2005 to 2016. We trained and cross validated our model using a k-fold cross-validation where *k=12*. For diabetes, obesity, and exercise, each year had 50 data points, one for each state (including the District of Columbia and excluding the state of Hawaii). In total, the training was performed using 600 data points. Note that we did not include data points from 2004 in our experiments. This is done to have a consistent training and test set as our proposed methods require a *lag* where data from 2004 is used in creating a feature vector for 2005, and similarly for following years.

We later tested each of our models trained on data from 2005 to 2016 on the years 2017 and 2018, and calculated the mean absolute error (MAE), root mean squared error (RMSE), symmetric mean absolute percentage error (SMAPE) [65], Spearman’s Correlation Coefficient (rho), and Pearson’s Correlation Coefficient (R).

We emphasize that, during development, we never looked at the results on the test set, and so none of our design decisions were influenced by them to safeguard against implicit overfitting. This is one of the key shortcomings of the previous literature, most of which employ in-sample evaluation.

### Alternate Approaches

We performed 6 different approaches for each of the 3 target variables for the US region. We have defined these 6 experiments in detail as follows.

#### Trivial Baseline

For evaluation purposes, we used the trivial *last year equals this year* baseline. With this in mind, we used 2016’s prevalence as a prediction for 2017, and 2017’s prevalence as a prediction for 2018. We, then, computed the MAE, RMSE, SMAPE, rho, and R, to be used as baseline for evaluation purposes.

#### Spatial Model

We extended the methodology presented in Nguyen et al’s paper [42] of applying a national spatial-only model to temporal dimension. This becomes our secondary baseline.

#### Spatio-Temporal Model

This experiment is based on our main methodological contribution where we used a corrective approach to first neutralize the effect of Google’s normalization as a preprocessing step, and, then, trained the model. We called it *Spatio-Temporal* to signify the use of both spatial and temporal Google Trends data.

#### Multivariate Model

To boost the performance of our spatio-temporal model, we used the *trivial baseline* as a covariate. More concretely, we extended our spatio-temporal model to use the actual prevalence of the previous year as an auxiliary feature to predict the prevalence of the current year. We called the extended model *multivariate* to signify the inclusion of the covariate.

#### Lagged Multivariate Model

While training the previous model, we made a crude assumption that the population search behavior for any particular year is correlated to the prevalence of that year. However, we realized that it may be possible for the search behavior of any year to be predictive of the next year. As an example, search behavior in 2017 may be more predictive of 2018 prevalence than of 2017. To test this theory, we experimented by shifting our time window for the multivariate regression.

#### Hierarchical Lagged Multivariate Model

One of the simplifying assumptions we made while training the previous models is that the predictive pattern of Google Trends is the same across all states. This assumption may, however, not be valid. In particular, each state might have a different base prevalence rate for the health condition being modeled. To incorporate subnational bias terms, we explicitly included the state ID as a covariate. We did this by extending the feature vector to include a one-hot vector of 50 states. By doing this, we implicitly modeled a hierarchical distribution where we considered national trends, as well as subnational trends.

### Transfer Learning

We tested the generalizability of our methodology across countries by conducting 2 further experiments for the prevalence of diabetes and obesity in Canada. We collected the Google Trends data for Canada in a similar fashion as we did for the United States. The offline health statistics for diabetes and obesity were collected from the Statistics Canada site [66]. One limitation pertaining to these statistics was that, starting from 2015, the collection strategy and the design of the sampling process for synthesizing statistics has changed, rendering pre-2015 data incomparable to post-2015. Therefore, we dealt with these 2 periods of data separately in our experiments. A second point to note is that diabetes and obesity statistics for Canada are not available for 2004 and 2006. We, therefore, collected the offline data from 2007 onward only. Owing to these 2 limitations and the need to have 1 separate year for the lagged models, our training set for Canada included data from 2008 to 2012 with each year containing 10 data points, 1 per Canadian province. We used data from 2013 and 2014 as a test set. Owing to the change in sampling methods and the limited number of years, we did not train a separate model for 2016-2018. However, we reported the results for these years as a test set.

#### Cross-Country Generalizability of the Method

In the first set of experiments in the context of transfer learning, we used the same set of experiments that we conducted for the United States, to make predictions for Canada.

#### Cross-Country Generalizability of the Model

For the second set of experiments, we trained our lagged multivariate model on the United States on 14 years (from 2005 to 2018) to test across Canada. The purpose for this experiment was 2-fold. First, we wanted to test the generalizability of our trained model across other *similar* countries by using Canada as a proxy. Second, we wanted to test the reliability of applying the models in such a fashion to other countries where offline data might not be available. We have briefly described the importance of this step later in the Discussion section.

## Results

### Evaluation

For the evaluation of our experiments, we measured the performance of different models over 5 metrics: MAE, RMSE, SMAPE [65], rho, and R. To compute the correlation coefficients, we concatenated the predictions across all the test years and used those to compute a global correlation.

To test for statistically significant improvements in the MAE, we conducted a one-sided paired *t* test across each set of experiments in relation to the trivial baseline. We also computed statistical tests for each extension such that the spatio-temporal model gets compared with the spatial model, the multivariate spatio-temporal model gets compared with the spatio-temporal model, and so on. We do this to evaluate the gain, if any, obtained by each extension.

### US Based Models

A detailed evaluation of our experiments on the United States can be found in Table 3. The best way to interpret the results is to read the values for each statistic from left to right.

**Table 3:** A detailed evaluation of 5 different experiments across the 3 target variables for the region of the United States.

Target variable		Trivial baseline	Spatial model	Spatio-temporal model	Multivariate spatio-temporal model	Lagged multivariate spatio-temporal model	Hierarchical lagged multivariate spatio-temporal model
**Diabetes**							
	MAE^a^	0.72	0.81	*0.72* ^b^	*0.65* ^c^	*0.62* ^c^	*0.55* ^c^
	RMSE^d^	0.92	1.0	*0.91*	*0.81* ^c^	*0.80* ^c^	*0.72* ^c^
	SMAPE^e^	6.94	7.63	*7.13*	*6.23* ^c^	*6.04* ^c^	*5.24* ^c^
	Spearman rho	0.87	*0.89* ^c^	0.87	*0.88* ^c^	*0.89* ^c^	*0.93* ^c^
	Pearson R	0.90	0.90	0.88	*0.91* ^c^	0.91^c^	*0.94* ^c^
**Obesity**							
	MAE	1.20	2.81	*2.09*	*1.24*	*0.99* ^c^	1.08^c^
	RMSE	1.55	3.28	*2.56*	*1.59*	*1.33* ^c^	1.40^c^
	SMAPE	3.88	9.31	*6.96*	*4.03*	*3.22* ^c^	3.51^c^
	Spearman rho	0.93	0.87	0.85	*0.94* ^c^	0.93	*0.95* ^c^
	Pearson	0.93	0.86	0.86	*0.94* ^c^	0.94^c^	*0.95* ^c^
**Exercise**							
	MAE	2.89	*2.32* ^c^	3.12	*2.47* ^c^	*2.36* ^c^	*1.95* ^c^
	RMSE	3.32	*2.75* ^c^	3.75	*2.90* ^c^	*2.83* ^c^	*2.40* ^c^
	SMAPE	3.85	*3.11* ^c^	4.11	*3.32* ^c^	*3.16* ^c^	*2.62* ^c^
	Spearman rho	0.68	*0.73* ^c^	*0.81* ^c^	0.71^c^	*0.77* ^c^	*0.80* ^c^
	Pearson R	0.69	*0.74* ^c^	*0.80* ^c^	0.72^c^	*0.78* ^c^	*0.81* ^c^

^a^MAE: mean absolute error.

bThe values in italics signify an improvement in the performance in comparison to the previous method.

^c^The method beat the trivial baseline.

^d^RMSE: root mean squared error.

^e^SMAPE: symmetric mean absolute percentage error.

Toward our first research objective to correct the methodological shortcomings of the previous literature, we developed a *spatio-temporal model* for nowcasting lifestyle diseases. We observed that the spatio-temporal model performs better than the spatial model for diabetes and obesity but not for exercise. The performance improvement over the spatial method for diabetes in terms of the MAE (0.81-0.72) and RMSE (0.91-0.81) was 11% (*P*=.06), whereas the improvement for obesity was 26% in MAE (2.81-2.09) and 22% in RMSE (3.28-2.56; *P*<.001). However, only the results for obesity are significant. Both of these improvements in MAE are statistically significant.

To improve upon the spatio-temporal model, we, then, trained a *multivariate spatio-temporal model*, by using the previous year’s prevalence as a covariate in combination with features from Google Trends. This improves the performance over the spatio-temporal model decreasing the error for diabetes by 10% in MAE (0.72-0.65) and 12% in RMSE (0.91-0.81), obesity by 41% in MAE (2.09-1.24) and 38% in RMSE (2.59-1.59), and exercise by 21% in MAE (3.12-2.47) and 23% in RMSE (3.75-2.90). Of these improvements, diabetes was not significant (*P*=.08), but both obesity and exercise were statistically significant (*P*<.001 and *P*=.001, respectively).

In the next set of experiments, we shifted our dependent response variable by 1 year to test for a time lag in the impact of search behavior on disease statistics. We called this the *lagged multivariate spatio-temporal model*. In comparison with the multivariate spatio-temporal model, this model improves performance on diabetes by 5% in MAE (0.65-0.62) and 1% in RMSE (0.81-0.80), on obesity by 20% in MAE (1.24-0.99) and 16% in RMSE (1.59-1.33), and on exercise by 4% in MAE (2.47-2.36) and 2% in RMSE (2.90-2.83). The improvement was statistically significant for diabetes and obesity (*P*=.02 and *P*<.001), but not for exercise (*P*=.17).

Finally, to account for subnational trends, we trained a *hierarchical* *lagged multivariate spatio-temporal model* where we included the state ID as a covariate. In comparison with the lagged model, we got a performance improvement on diabetes and exercise, but a decrease in performance on obesity. For diabetes, the MAE improves by 11% (0.62-0.55) and RMSE by 10% (0.80-0.72). For exercise, the MAE improves by 17% (2.36-1.95) and RMSE by 15% (2.83-2.40). Whereas for obesity, the performance deteriorates by 8% in MAE (0.99-1.08) and by 5% in RMSE (1.33-1.40). For both diabetes and exercise, the performance improvement was statistically significant (*P*=.01 and *P*=.02).

In terms of the overall improvement over the trivial baseline, our best models (ie, hierarchical lagged multivariate spatio-temporal model for diabetes, and exercise, and lagged multivariate spatio-temporal model for obesity) result in 24% improvement in MAE (0.72-0.55) and a 22% improvement in RMSE (0.92-0.72) for diabetes; 18% improvement in MAE (1.20-0.99) and a 14% improvement in RMSE (1.55-1.33) for obesity, and 34% improvement in MAE (2.89-1.95) and a 28% improvement in RMSE (3.3-2.40) for exercise. All of these improvements are found to be statistically significant (*P*<.001 for diabetes, *P*=.001 for obesity, and *P*<.001 for exercise). The SMAPE, Spearman rho, and Pearson R follow similar trends.

### Transfer Learning

For the experiments for our transfer learning framework, we evaluated (1) cross-country generalizability of the method (training and evaluating models just for Canada) and (2) cross-country generalizability of the model (taking a trained-on-US model and evaluating it for Canada). We summarized the results achieved for each of the proposed methods in Tables 4-6.

Whereas for the US case, most of our methods beat the trivial baseline, this is not the case for either of the 2 transfer settings. For the approach of retraining a Canada-specific model, we hypothesized that this is due to data scarcity with fewer data points for a given year (10 vs 50) and fewer years to train on (2008-2012 vs 2005-2016) being available compared with the US case. For some of the experiments (eg, diabetes model trained on 2008-2014 and tested on 2016-2018) where we beat the baseline, the improvements were only marginal. A detailed set of evaluation can be found in Tables 4 and 5.

Although the approach of applying the trained-on-US model to Canada fails to beat the trivial this-year-is-same-as-last-year baseline, we expect the highest utility of such approaches to be reliable when health statistics for the target country are unavailable. For this scenario, our results showed promise by achieving an average Spearman and Pearson correlation of 0.70 for diabetes, and 0.91 and 0.92 for obesity by using a pretrained model of a *similar* country (which, in our case, is the United States). We believe that these results are encouraging and worth replicating for developing countries.

Table 6 shows the results for the transfer learning framework across the 2 target variables for Canada for generalizability of the US trained model. We report the performance of the US-based model trained for the years 2005 to 2018 on Canada for the test years 2008-2014. We separately also report the performance of the model on the years 2016-2018 and 2012-2013 for the readers to compare the performance of the model trained on the United States to the model trained on Canada, both tested on the same test set.

**Table 4:** Results for the transfer learning framework across the 2 target variables for Canada for generalizability of the method trained over the years 2008-2012, and tested on the years 2013 and 2014.

Target variable		Trivial baseline	Spatial	Spatio-temporal model	Multivariate spatio-temporal model	Lagged multivariate spatio-temporal model	Hierarchical lagged multivariate spatio-temporal model
**Diabetes**							
	MAE^a^	0.54	0.80	0.83	*0.70* ^b^	*0.66*	*0.61*
	RMSE^c^	0.66	0.96	1.00	*0.86*	*0.81*	*0.73*
	SMAPE^d^	7.64	11.57	12.10	*10.18*	*9.64*	*8.64*
	Spearman	0.86	0.68	0.62	*0.72*	*0.76*	*0.79*
	Pearson	0.85	0.64	0.59	*0.74*	*0.76*	*0.81*
**Obesity**							
	MAE	1.31	1.68	1.74	*1.57*	1.59	1.81
	RMSE	1.66	2.56	2.80	*2.02*	*1.87*	2.45
	SMAPE	5.81	7.31	8.05	*7.24*	*6.92*	7.88
	Spearman	0.89	0.82	*0.85*	*0.87*	*0.89*	0.85
	Pearson	0.95	0.86	0.86	*0.95*	*0.95*	0.91

^a^MAE: mean absolute error.

^b^The values in *italics* signify an improvement in the performance in comparison to the previous method.

^c^RMSE: root mean squared error.

^d^SMAPE: symmetric mean absolute percentage error.

**Table 5:** Results for the transfer learning framework across the 2 target variables for Canada for generalizability of the method trained over the years 2008-2014, and tested over the years 2016-2018.

Target variable		Trivial Baseline	Spatial	Spatio-temporal model	Multivariate spatio-temporal model	Lagged multivariate spatio-temporal model	Hierarchical lagged multivariate spatio-temporal model
**Diabetes**							
	MAE^a^	0.84	0.82	*0.75* ^b,c^	*0.71* ^c^	*0.68* ^c^	0.74^c^
	RMSE^d^	1.07	*1.04* ^c^	*0.93* ^c^	0.93^c^	*0.86* ^c^	0.99^c^
	SMAPE^e^	11.16	*10.74* ^c^	*9.78* ^c^	*9.37* ^c^	*9.00* ^c^	10.05^c^
	Spearman	0.69	*0.82* ^c^	*0.84* ^c^	0.76^c^	*0.78* ^c^	0.78^c^
	Pearson	0.7	*0.80* ^c^	*0.82* ^c^	0.76^c^	*0.77* ^c^	*0.78* ^c^
**Obesity**							
	MAE	1.57	7.98	8.72	*3.39*	*2.62*	5.69
	RMSE	2.37	8.4	9	*3.86*	*3.38*	5.92
	SMAPE	4.99	29.59	33.59	*11.73*	*8.36*	20.71
	Spearman	0.93	0.86	*0.89*	*0.92*	*0.93*	*0.95* ^c^
	Pearson	0.91	0.88	*0.9*	*0.93* ^c^	0.91	*0.95* ^c^

^a^MAE: mean absolute error.

^b^The values in *italics* signify an improvement in the performance in comparison to the previous method.

^c^The method beat the trivial baseline.

^d^RMSE: root mean squared error.

^e^SMAPE: symmetric mean absolute percentage error.

**Table 6:** The results for the transfer learning framework across the 2 target variables for Canada for generalizability of the US trained model.

Cross-country generalizability of the US-based model			Diabetes				Obesity		
			Trivial baseline		Lagged multivariate model		Trivial baseline		Lagged multivariate model
**Train years: 2005-2018 (US); test years: 2008-2014 (Canada)**									
	MAE^a^	0.68		0.88		1.53		1.78	
	RMSE^b^	0.92		1.10		1.96		2.16	
	SMAPE^c^	9.9		12.65		6.91		8.20	
	Spearman	0.81		0.77		0.90		0.90	
	Pearson	0.76		0.74		0.91		0.91	
**Train years: 2005-2018 (US); test years: 2016-2018 (Canada)**									
	MAE	0.84		1.29		1.57		*1.54*	
	RMSE	1.07		1.49		2.37		*2.25*	
	SMAPE	11.16		16.04		4.99		*4.92*	
	Spearman	0.69		0.59		0.93		0.92	
	Pearson	0.70		0.60		0.91		0.91	
**Train years: 2005-2018 (US); test years: 2013-2014 (Canada)**									
	MAE	0.54		0.91		1.31		1.31	
	RMSE	0.66		1.12		1.66		*1.60* ^d^	
	SMAPE	7.64		13.10		5.81		*5.76*	
	Spearman	0.86		0.74		0.89		*0.90*	
	Pearson	0.85		0.74		0.95		0.95	

^a^MAE: mean absolute error.

^b^RMSE: root mean squared error.

^c^SMAPE: symmetric mean absolute percentage error.

^d^The values in italics signify improvement in performance over the trivial baseline.

## Discussion

### Principal Findings and Contributions

#### Value and Validity of Modeling Noncommunicable Diseases

The *slow-moving* nature of NCDs compared with the relatively faster moving trends in search behavior makes it a hard problem to perform lifestyle disease surveillance using Google Trends. The background literature reports overly optimistic results in this arena. This is potentially a consequence of what is known as the *positive result bias* [67] in the scientific community, leading to claims that *most* published research findings may be false [68,69]. We show in this work that in addition to the methodological shortcomings, *none* of the previous studies compares its results to the trivial *last year equals this year* baselines, which, surprisingly, is hard to beat. In this study, we empirically test the feasibility of the task by experimenting across different methods for 3 target variables. Our experiments are ordinal in nature as each subsequent experiment is an extension of the previous one. Although most of our latter extensions beat the trivial baseline, modeling NCDs is not a trivial problem. The main challenge of the problem lies in the scarcity of ground truth data, which is typically only available on an annual basis. Even if the data were available on a finer granularity, the inherent nature of NCDs, such as diabetes and obesity, does not allow for discernible monthly or weekly variation. Even the relative year-to-year changes are low which is why the correlation coefficients for the trivial baselines are high, and hard to beat. As a result, we anticipate a low-to-moderate value in modeling the *estimation* of NCDs, as well as in the validity of Google Trends for nowcasting lifestyle diseases.

The lack of validity of Google Trends in the given context can be partly attributed to changes in Web search behavior across time. As an example, Web search users might long have realized the potential to use Google for navigational queries, instead of having to remember and type exact website URLs. However, the use for informational queries in the health domains is likely to be still growing, also as Google adds new features, trying to answer common health-related questions directly on the search result page. A related but similar reason is that the meaning and perception of terms themselves can change over time. In linguistics, this phenomenon is known as the *semantic shift* [70] describing how the senses of words drift over time. A typical example of that is the word *Gay* which evolved from its meaning of *lighthearted* and *joyous* in the 1900s to *homosexual* in the 1990s [71]. With increasing popularity of social media sites and Web-based tools, Web-based content is being produced expeditiously, leading to relatively frequent semantic shifts. Accounting for these complex phenomena with the scarcity of data is already a difficult problem. Additional limitations of the Google Trends framework make it an even challenging issue. These limitations include the changes in geolocation assignment applied to Google Trends in 2011, Google Trends’ normalization scheme, and, finally, instability of search indices of a given keyword on different days [31,57].

As for the first limitation, in 2011, Google implemented significant improvements in the geolocation of search queries (a note on the Web interface for Google Trends says: “An improvement to our geographical assignment was applied from 1/1/2011.”) To account for these changes, in the earlier set of experiments for this study (not reported in this manuscript), we limited our training set to data from 2011 onward only. Unfortunately, none of our experimental models beat the trivial baseline. In the current set of experiments, we include the years from before 2011 to boost the performance, assuming that the geolocation assignment at the state level was not affected by the changes. We observe that this, in fact, is true and that more data helps learn better models. On the surface, this is not an interesting finding as machine learning models are inherently data hungry. However, we hypothesize that the boost in performance may also be attributed to learning semantic and usage shifts in data. By including a wider time window, we believe our models may implicitly be selecting features that are shift-independent, and pruning out features that are not. For example, the keyword *slim*, which was part of our obesity-related models, was assigned a weight of zero. Since Google Trends provides related topics for each keyword, entering the keyword *slim* yields topics such as *slim-fit pants* and *Plexus* along with *Xbox-Console* and *PlayStation 3*. This indicates that the keyword *slim* is used in 2 different contexts: obesity and slim console games. There was a potential semantic shift after 2010 when Xbox 360 slim model was released [72], and it is possible that our model was able to capture it given a wider time window, resulting in pruning it out.

A final limitation of Google Trends in the context of this study is the instability of search indices. We observe that repeatedly asking for the same data from Google Trends’ can return different results. To correct for this, like [31], we resolve potential data instability issues by averaging data points collected across 10 consecutive days for the United States, and across 3 consecutive days for Canada.

#### Model Selection and Learning

**Figure 2:**
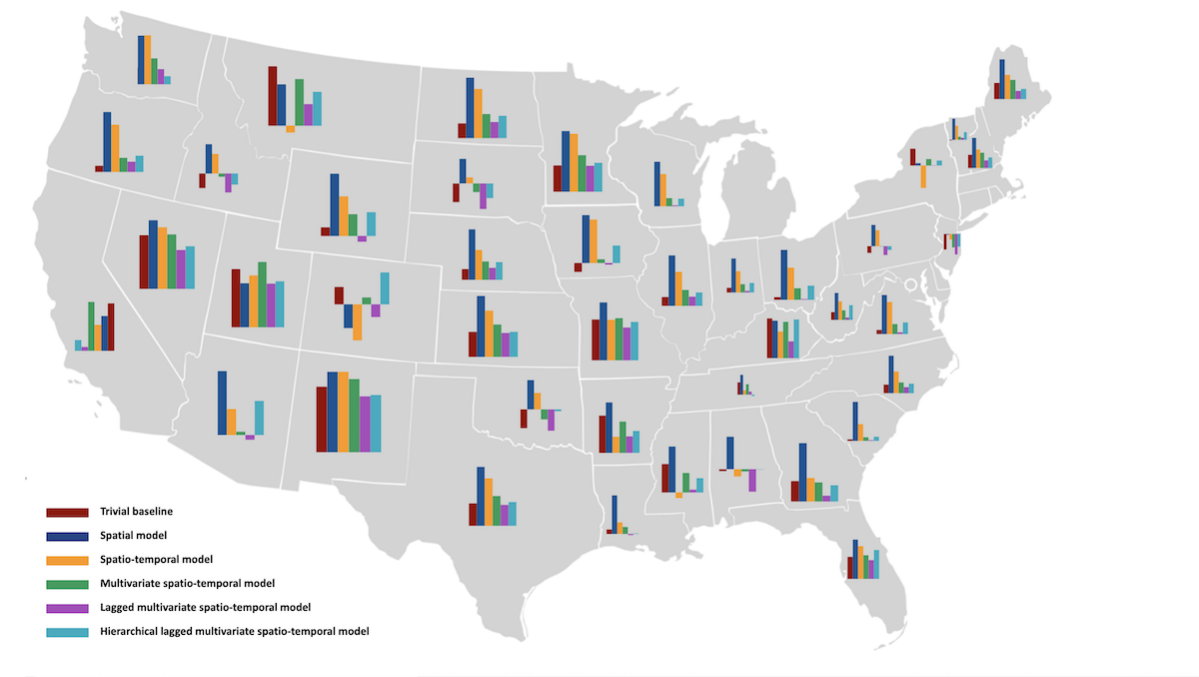
The comparison of the errors of 6 different methods for the target variable obesity for the year 2018 for each state. (Note: The bars represent the simple error [ie, ground truth prediction] for each state, and not the predicted diabetes or obesity rates. Therefore, the height of the bars is only comparable within each state and not comparable across states as the scale is not fixed. As most bars are above zero, this indicates that in most cases, the models underestimate the ground truth obesity rates.).

In our experiments, we observe that a time-lagged multivariate model, which uses both ground truth and search data from the past year to nowcast the current year, improves over our previous, simpler methods. This suggests that the implications of the search behavior are observed in the NCD prevalence of later year rather than the immediate one. A final extension to our models is the use of state-ID as a covariate to model a hierarchical distribution. The inclusion of these state-level offsets significantly improves the results for diabetes and exercise. One interesting observation is that almost all of our models seem to underestimate the rate of NCDs. This is apparent in Figure 2, which shows how the models consistently underestimate the prevalence of obesity. We observe the same behavior across the other target variables. A potential reason is that of users moving away from the keywords used in this study as a consequence of a change in search behavior. This ties back to the discussion on the semantic and search behavior changes of users across time.

#### Geographical Transfer Learning

This work is one of the very few to test the geographical generalizability of the proposed method. We use Canada as a test case as it is close to the United States, and is one of the very few English-speaking countries to have reliable *state-level* statistics. On the basis of our evaluation, most of the models trained on Canada do not beat the baseline. This can again be attributed to the scarcity of data as in comparison with the United States, Canada has only 10 provinces and the usable data are only available starting 2007. Despite the failure to beat the baseline, we see opportunities for public health monitoring in countries where the public health monitoring system is less developed and so the otherwise trivial baseline cannot be applied. In particular, it seems feasible to monitor relative across-state or across-year trends, rather than absolute prevalence rates. Extending our approach to countries with low resources comes with certain challenges though as, exactly due to their lack of trustworthy data, it is hard to have an objective evaluation of the system performance.

#### Proactivity Versus Reactivity

In terms of the health-related behavior, the user search behavior can be weakly classified into 2 categories: proactive and reactive. Proactive search behavior is when the search is derived from curiosity and awareness. This usually defines users who are cautious about their health with an interest in preventing a health condition. Reactive, on the other hand, defines behavior, which is corrective where users are seeking help to treat or manage their health problems. In the case of diabetes and obesity in the United States and Canada, we observe that predictive search terms indicate a highly reactive behavior, with *diabetes symptoms, diabetic diet, and exercise* carrying higher absolute weights (and, hence, higher significance). This potentially indicates that it is mostly users affected by a condition who show interest. If this interpretation holds, this points toward a need to carry out public health interventions to try to change the information seeking to be more proactive.

#### Keyword Selection Using Google Correlate

For our study, we tried to limit the risk of overfitting by following a robust keyword selection process both in the preprocessing phase as well as the modeling phase. As a preprocessing step, we used Semantic-Link, Google Trends–related keywords, and Google Correlate as tools to select the best set of features. This is one of the key strengths of our study where we combine semantically connected, cooccurring, and correlated terms to create a diverse keyword list. In the context of NCDs, this is the first study to utilize the strength of Google Correlate in the process of keyword selection. An interesting case study in this context is that of obesity-related keywords, a subset of which is shown in Table 2. One of the top keywords is *dresses plus size*, which is also included in the L1 regularized model. Although it is a fairly intuitive keyword in hindsight, it would have been challenging to discover it without Google Correlate. Although we do not perform an in-depth analysis of the keywords in this study, we encourage researchers to use pseudo bootstrapping approaches in their keyword selection process to perform richer analysis. In terms of Google Correlate, unfortunately, a note on the Web interface says: “Google Correlate will shut down on December 15th 2019 as a result of low usage,” as a result of which it will not be available for future researchers.

### Limitations

Currently, our approach is agnostic to word semantics and we just explore what is predictive of a particular NCD without accounting for anomalies. In this context, recent methods on the use of word embeddings may be useful to curb inconsistent patterns of associations. It would also be useful if we can explicitly capture the semantic or behavior shifts in time, and make it part of the model. This warrants use of sophisticated dynamic network analysis tools and other time-series models.

One more potential limitation of this study is the inability to test our methodology on other countries. Although we show promising results for Canada, it would have been preferable to test our methods to other English, and even non-English–speaking countries. This limitation was primarily a consequence of the unavailability of state-level ground truth for many countries.

Finally, one limitation pertaining to our data collection process is that while like [31], we resolve potential data instability issues by averaging data points collected across 10 consecutive days for the United States, we use only 3 data points for Canada. We do this in the interest of time, as the experiments for Canada are more preliminary. Nonetheless, to get a sense of whether variation between days might affect the overall conclusions, we conducted a day-to-day test-retest reliability analysis across 3 days from Google Trends data for Canada, using Spearman and Pearson correlation. We computed the correlation coefficient between each set of data collected on day 1 and day 2, and between data collected on day 2 and day 3. For day 1 and day 2, we found an average Spearman and Pearson correlation of 0.85 and 0.86 for temporal data, and of 0.84 and 0.88 for the spatial data. For day 2 and day 3, we found an average Spearman and Pearson correlation of 0.85 and 0.86 for temporal data, and of 0.84 and 0.89 for the spatial data. Given that (1) the day-to-day correlations are very high, and that (2) we nevertheless average across 3 days, we do not expect our conclusions for Canada to be affected by using data points from only 3 rather than 10 days.

### Future Research Directions

Using Google Trends for *nowcasting or estimating* the spatio-temporal prevalence of NCDs may not always be of significant value due to the scarcity of data, uncertainty of search behavior, and the changes in usage of different keywords across time. However, there are still several fronts that remain unexplored.

In terms of the NCDs, using Google Trends for *qualitative* research is an overlooked territory. In this context, feature analysis is a promising venue where tracking, investigating, and discovering different keywords could help policy makers or governments make better decisions.

In terms of the qualitative analysis, it might also be useful to employ Google Trends for analyzing NCD-related *events*. In this context, events could be described as short-term (or single-day) phenomenon. A popular example of that is initiating and monitoring public health campaigns and interventions. For health campaigns, it is useful to conduct both prehoc and posthoc analyses. Prehoc analyses include determining the target audience, and baseline search behaviors of people. On the other hand, posthoc analyses characterize changes in the search behavior triggered by the target event. Examples of related work in the context of public health awareness campaigns can be found in [73-76].

Another promising future direction includes the use of task-specific modeling techniques where the domain knowledge about the task at hand could be used to craft better machine learning models. In our study, we use the state-ID as a proxy to model hierarchical distribution with different country-level and state-level behaviors. However, our models are still rudimentary and one could potentially employ more sophisticated models, such as hierarchical linear models [77], or neural regression trees [78], to model different hierarchies. In our study, we model the hierarchy based on the state-ID. The state, in this case, is treated as a *cluster* selected a priori to the modeling based on the domain knowledge. One could also try to find optimal clusters (such as group of similar states) or partitioning of the data simultaneously while modeling the problem itself. This can be achieved by using techniques such as nearest neighbors [79] or other tree-based approaches such as those shown in [78,80]. An added advantage of the hierarchical models is their increased interpretability resulting in some qualitative insights. Such models also allow us to optimize feature selection on subnational levels while modeling a national distribution, improving the overall robustness. Clustering approaches could also lead to *directed* public health interventions by providing cluster-specific insights. Governmental public health interventions would be much more effective if they target the right audience, and in a right and directed manner, taking into account the local search behavior.

In summary, we believe that despite limited promise for quantitative NCD surveillance, Google Trends still holds value in relation to NCDs, particularly for qualitative analyses or for monitoring the effect of public health campaigns. Finally, in terms of other fast-moving trends, any useful spatio-temporal national-level surveillance using Google Trends necessitates the use of appropriate denormalization such as the one shown in this work.

### Conclusion

In this paper, we first review the methods presented in the background literature. We present a comprehensive table where we compare the literature across different metrics. One of the surprising findings of our study is the absence of evaluation against trivial baselines in all of the reviewed papers. We furthermore, highlight the methodological shortcomings of the most relevant research papers. In the second part of the paper, we use a corrective approach to improve upon the background work. Specifically, we explore the feasibility of using Google Trends for nowcasting the prevalence of lifestyle diseases in the context of diabetes, obesity, and exercise. To undo the effect of Google Trends’ normalization, we propose a novel spatio-temporal denormalization scheme. That combined with the trivial baseline as a covariate beat the previously set baseline methods in the background literature for most of the target variables. We further improve upon that model by shifting the time window by 1 year, and then including a state-ID as a covariate. Our best models beat the trivial baseline with a significant improvement in performance. We, however, realize that this requires both abundant data, as well as creative modeling strategies. Furthermore, we extend upon our formative work to show generalizability of our methodology and trained models in the international setting, setting a cornerstone for using such transfer learning-based approaches in low-resource countries. Finally, we propose various possible future paths researchers can take to conduct both quantitative and qualitative analyses using Google Trends.

## Appendix

Multimedia Appendix 1 The final postpruning keywords used in the modeling of each of the different target variables.

Multimedia Appendix 2 Supplementary information on Google Trends’ denormalization procedure.

## Abbreviations

BRFSS: Behavioral Risk Factor Surveillance System
GFT: Google Flu Trend
ILI: influenza-like illness
MAE: mean absolute error
NCDs: noncommunicable diseases
RMSE: root mean squared error
SMAPE: symmetric mean absolute percentage error
STI: sexually transmitted infection
