# Supplementary material for: Lifestyle Disease Surveillance Using Population Search Behavior: Feasibility Study
Source: J Med Internet Res. 2020 Jan 27;22(1):e13347. doi: 10.2196/13347 (PMC7011125; doi:10.2196/13347)
Supplement: Multimedia Appendix 1 [file jmir_v22i1e13347_app1.docx]

## Multimedia Appendix 1

Table 6. This table shows the final post-pruning keywords used in the modeling of each of the different target variables.

| **Diabetes** | **Obesity** | **Exercise** |
| --- | --- | --- |
| diabetic | gastric | yoga |
| diabetic diet | diabetic | ejercicios |
| diabetes insulin | dresses plus size | bodybuilding |
| diabetes mellitus | symptoms of high blood sugar | gym |
| signs of diabetes | obese | bike repair |
| insulin syringes | symptoms of congestive heart failure | bike helmet |
| sugar level | nutrition | jogging |
| endocrine | inactivity | bike laws |
| NIH | weighing | fitness |
| cholesterol | weight loss | e-bike |
| ketoacidosis | visceral | bike locks |
| type 2 diabetes | unhealthy | iPod |
| sclerosis | meals | bike sale |
| obesity | malnutrition | how to exercise |
| icd 10 codes | insulin | bodybuilding |
| hypertension | how to lose weight |  |
| hyperglycemia | healthy | pre workout |
| glucose | exercise | gym near me |
| diabetic ketoacidosis | diet | fitness gym |
| diabetes | apnea | exercise |
| insulin | abdominal | calories |
| polyphagia | dietary | aerobic exercise |
| symptoms of diabetes | slim | workout |
| prediabetes | pizza delivery | trainer |
| diabetes insipidus | wellness | ipod reset |
| diabetes symptoms | calories | exercises |
| type 2 | cholesterol | quinoa gluten free |
| glycogen |  | best workout |

## 
