# Supplementary material for: Lifestyle Disease Surveillance Using Population Search Behavior: Feasibility Study
Source: J Med Internet Res. 2020 Jan 27;22(1):e13347. doi: 10.2196/13347 (PMC7011125; doi:10.2196/13347)
Supplement: Multimedia Appendix 2 [file jmir_v22i1e13347_app2.pdf]

Google Trends: Normalization & De-Normalization

Shahan A. Memon*, Saquib Razak*, Ingmar Weber+
* Carnegie Mellon University
+ Qatar Computing Research Institute

Google Trends

Google Trends

[..] shows how often a particular search-term is entered relative to the total search-volume across various regions of the world, (for different time periods starting 2004), and in various languages.[1]

Google Trends

primarily shows two kinds of data:

Temporal (time-series), and Spatial (location-specific)

Temporal versus Spatial Data

Figure below shows **temporal** as well as **spatial** search intensity for the term **“diabetes”** in the **United States** for the time period **2004-present**

Temporal Data {

Spatial Data {

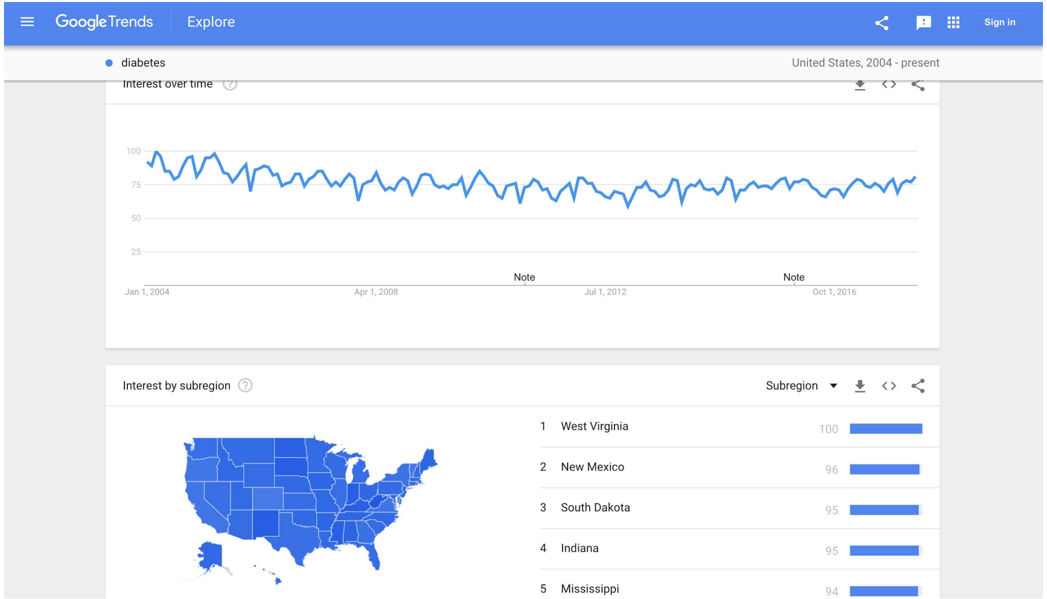

Google Trends: Normalization

Google Trends

normalizes data across time and space.

Temporal Normalization

Google returns data such that the period with the highest relative search intensity (circled below) corresponds to an arbitrary reference value of 100. All other temporal units are normalized with respect to this absolute maximum of 100.

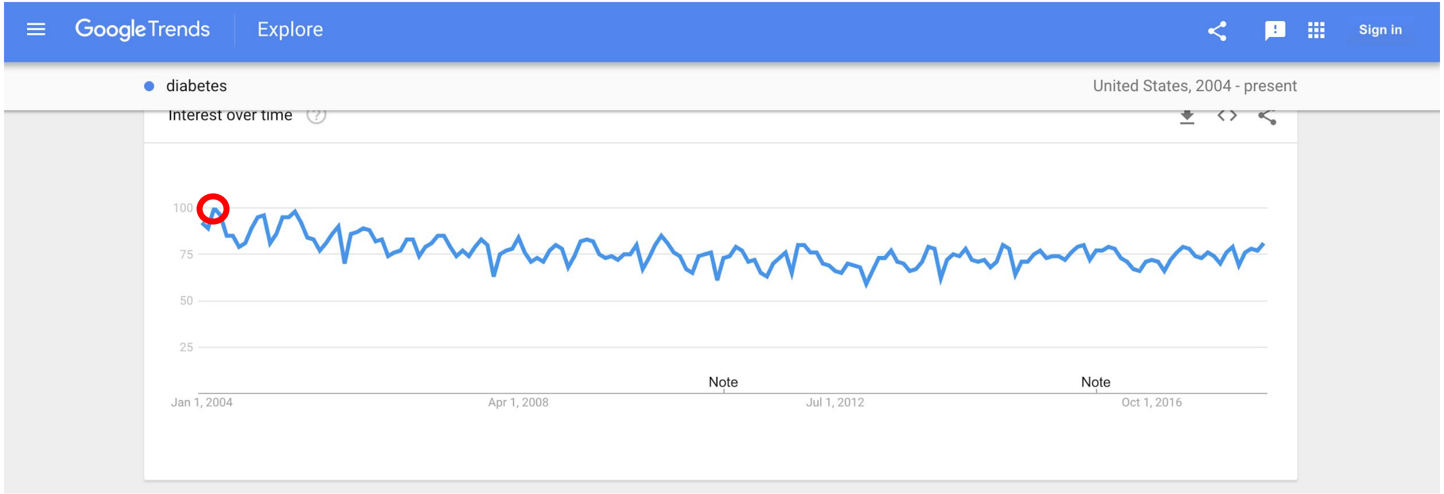

Spatial Normalization

Similarly, when getting search data across spatial units such as U.S. states, the state with the highest search intensity (circled below) is assigned a value of 100, and all other spatial units are normalized relative to the search intensity of that peak.

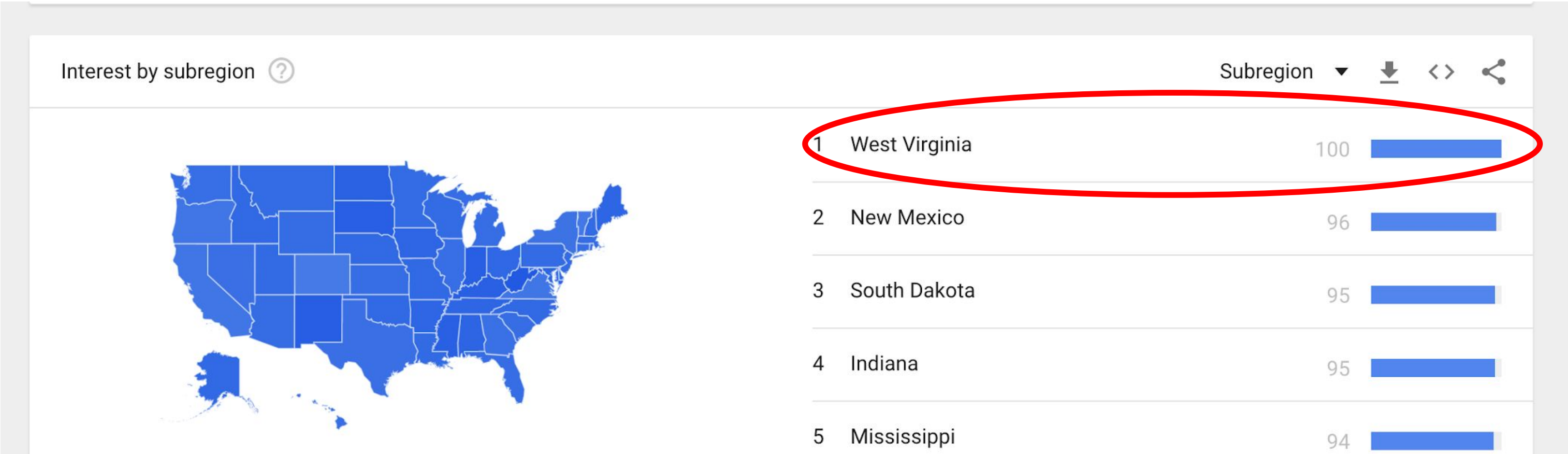

Consequences of

Google Trends Normalization

Characterizing a global slow-moving trend

Unlike fast-moving phenomenon such as influenza, stock markets, etc., slow-moving trends are trends that yield sparse temporal resolution (i.e. mostly measured across months, seasons or years). Examples: Rates of Diabetes, Obesity, Unemployment, etc.

- Fitting a global temporal-only model using Google Trends is hence infeasible due to the sparse set of data points (at max equal to number of years from 2004 to present)

Solution: Fit a spatial model to apply across time?
Example: Fit a model by using data points across each state and across each year to predict next year's trend

Modeling temporal variation using spatial-only data

- Spatial data alone **does not** reveal information about the changes in the **overall search volume**.
    - For example, if the global search volume for the United States was to go up from 2014 to 2015 with everything else the same (i.e. with ranking of states being the same), spatial data alone would not pick up that temporal trend. In fact it would treat 2014 exactly as 2015.

Modeling temporal variation using spatial-only data

- Moreover, within each year across space, data is normalized independently.
  - Hence, the value of 100 in year 2014 for the state New Mexico cannot be compared to the value of 100 in the year 2015 for the state of Mississippi.

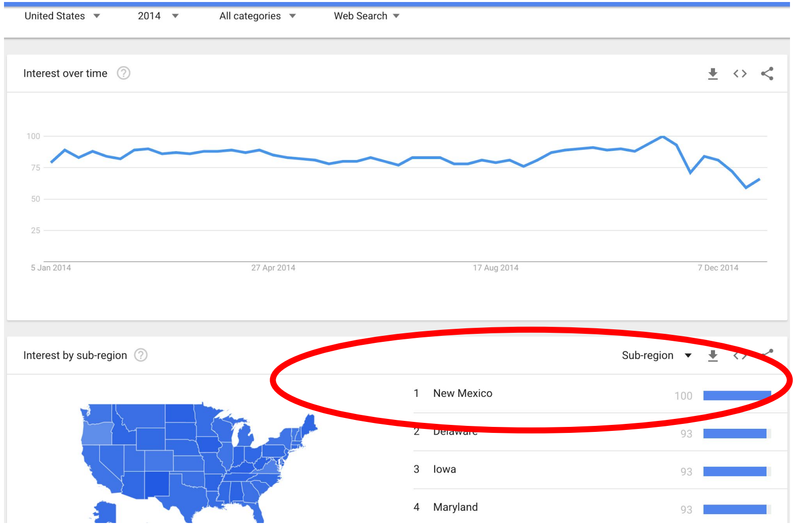

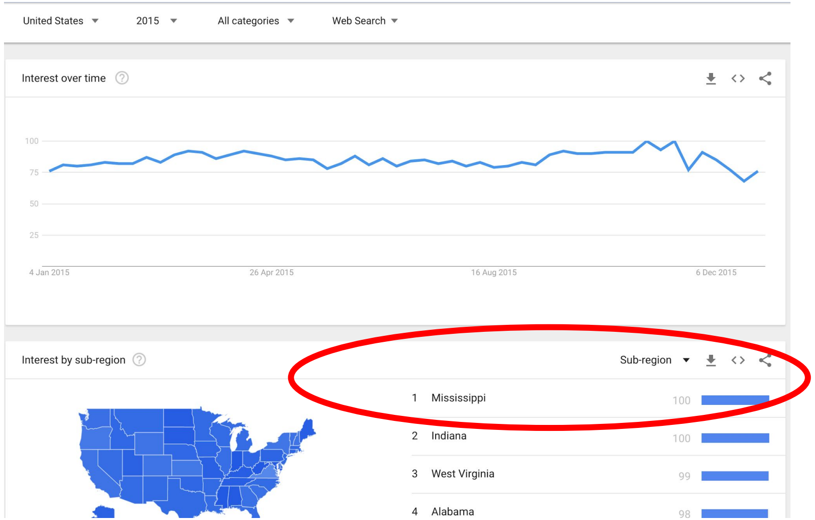

Modeling temporal variation using ~~spatial only~~ spatio-temporal data

Reconstructing the state-level contribution to the global value:

- ~~Solution 1:~~ Using actual absolute search volume from each of the states - ABSOLUTE SEARCH VOLUME NOT AVAILABLE

- Solution 2: Approximate the absolute search volume by creating a spatio-temporal index for each state by de-normalizing Google Trends data

Google Trends:
De-Normalization

Google Trends: De-Normalization (steps)

1. Collect Spatial as well as Temporal data
2. Choose a reference year r
3. Rescale each value using the following formula:

$$\hat{x}_{ys} = G_l(x_{ys}) * \frac{G_t(z_y)}{G_t(z_r)} * \frac{\sum_i^n G_l(x_{ri})}{\sum_i^n G_l(x_{yi})}$$

- New Spatio-Temporal Index
- Spatial Data value from Google Trends
- Ratio of Temporal Increase from year r to year y
- Ratio of the sum of spatial data in year r to that of year y

Terminology:

- G signifies that the data was collected via Google Trends.
- G_l signifies spatial data.
- y represents the year.
- n represents the number of states.
- r represents the reference year.
- $G_t(z_y)$ represents the value of the corresponding keyword in year y across time.
- $G_t(z_r)$ represents the value of the corresponding keyword in reference year r across time.
- $\sum_i^n G_l(x_{ri})$ represents the sum of the regional distribution of the corresponding keyword in the reference year r.
- $\sum_i^n G_l(x_{yi})$ represents the sum of the regional distribution of the corresponding keyword in the year y.

Google Trends: De-Normalization (fictitious example)

1. Imagine a country "Utopia" with three states: Hogsmeade, Metropolis and La la land.
2. Imagine the Google Trends spatial and temporal data looks as follows with reference year 2011:

States	2011	2012	2013
Hogsmeade	48.3	86.4	100
Metropolis	100	100	80.3
La La Land	64.5	12.3	17.8
Utopia	44.9	72.5	100

Spatial Data

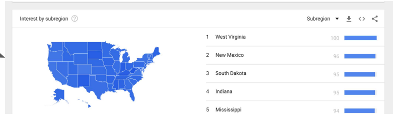

Temporal Data

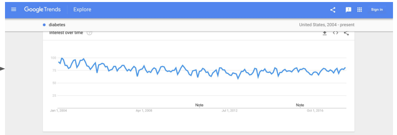

Google Trends: De-Normalization (fictitious example)

Since the values are normalized, so then absolute search volume would be equal to:

States	2011	2012	2013
Hogsmeade	48.3a	86.4b	100c
Metropolis	100a	100b	80.3c
La La Land	64.5a	12.3b	17.8c
Utopia	44.9u	72.5u	100u

where a, b, c and u are hidden from us.

2011: 48.3a + 100a + 64.5a = 44.9u
2012: 86.4b + 100b + 12.3b = 72.5u
2013: 100c + 80.35c + 17.85c = 100u

↓

2011: a (48.3 + 100 + 64.5) = 44.9u
2012: b (86.4 + 100 + 12.3) = 72.5u
2013: c (100 + 80.35 + 17.85) = 100u

↓

2011: $a \sum_i^n G_l(S_{r=2011,i}) = G_t(z_{r=2011}) * u$

2012: $b \sum_i^n G_l(S_{y=2012,i}) = G_t(z_{y=2012}) * u$

2013: $c \sum_i^n G_l(S_{y=2013,i}) = G_t(z_{y=2012}) * u$

Google Trends: De-Normalization (fictitious example)

Since the values are normalized, so then absolute search volume would be equal to:

States	2011	2012	2013
Hogsmeade	48.3a	86.4b	100c
Metropolis	100a	100b	80.3c
La La Land	64.5a	12.3b	17.8c
Utopia	44.9u	72.5u	100u

2011: 48.3a + 100a + 64.5a = 44.9u
2012: 86.4b + 100b + 12.3b = 72.5u
2013: 100c + 80.35c + 17.85c = 100u

↓

2011: a (48.3 + 100 + 64.5) = 44.9u
2012: b (86.4 + 100 + 12.3) = 72.5u
2013: c (100 + 80.35 + 17.85) = 100u

↓

2011: $a \sum_i^n G_l(S_{r=2011,i}) = G_t(z_{r=2011}) * u$
2012: $b \sum_i^n G_l(S_{y=2012,i}) = G_t(z_{y=2012}) * u$
2013: $c \sum_i^n G_l(S_{y=2013,i}) = G_t(z_{y=2012}) * u$

$a = (G_t(z_{r=2011}) * u) / (\sum_i^n G_l(S_{r=2011,i}))$
$b = (G_t(z_{y=2012}) * u) / (\sum_i^n G_l(S_{y=2012,i}))$
$c = (G_t(z_{y=2012}) * u) / (\sum_i^n G_l(S_{y=2013,i}))$

Google Trends: De-Normalization (fictitious example)

$a = (G_t(z_{r=2011}) * u) / (\sum_i^n G_l(S_{r=2011,i}))$
$b = (G_t(z_{y=2012}) * u) / (\sum_i^n G_l(S_{y=2012,i}))$
$c = (G_t(z_{y=2012}) * u) / (\sum_i^n G_l(S_{y=2013,i}))$

Note that according to our formula, if we now want to denormalize w.r.t to the reference year 2011, so by using our equation,

$$\hat{x}_{ys} = G_l(x_{ys}) * \frac{G_t(z_y)}{G_t(z_r)} * \frac{\sum_i^n G_l(x_{ri})}{\sum_i^n G_l(x_{yi})}$$

== 1/a as 2011 is our example reference year

== a or b or c depending on the year of the value being denormalized

States	2011	2012	2013
Hogsmeade	48.3	86.4	100
Metropolis	100	100	80.3
La La Land	64.5	12.3	17.8

↓

States	2011	2012	2013
Hogsmeade	48.3 a/a	86.4 b/a	100 c/a
Metropolis	100 a/a	100 b/a	80.3 c/a
La La Land	64.5 a/a	12.3 b/a	17.8 c/a

Google Trends: De-Normalization (fictitious example)

By applying our de-normalization method, our indices are hence off by a factor of “a”

Absolute Search Volume (Hidden)

States	2011	2012	2013
Hogsmeade	48.3 a	86.4 b	100 c
Metropolis	100 a	100 b	80.3 c
La La Land	64.5 a	12.3 b	17.8 c

Approximated Absolute Search Volume

States	2011	2012	2013
Hogsmeade	48.3 a/a	86.4 b/a	100 c/a
Metropolis	100 a/a	100 b/a	80.3 c/a
La La Land	64.5 a/a	12.3 b/a	17.8 c/a

De-Normalization (accounting for population and internet penetration)

Finally, to de-bias data of the population level, we would need to adjust each value by a product of the population in each state multiplied by the Internet penetration to get an approximate number of the Google search users in each state:

$$\hat{x}_{ys} = G_l(x_{ys}) * \frac{G_t(z_y)}{G_t(z_r)} * \frac{\sum_i^n G_l(x_{ri}) * P_{ri} * I_{ri}}{\sum_i^n G_l(x_{yi}) * P_{yi} * I_{yi}}$$

New Spatio-Temporal Index

Spatial Data value from Google Trends

Ratio of Temporal Increase from year r to year y

Ratio of the sum of spatial data in year r to that of year y

Ratio of population size of state n for the reference year r to the year y

Ratio of internet penetration of state n for the reference year r to the year y

Questions?

Email: SHAHAN A. MEMON at
samemon@cs.cmu.edu
